# P-95. Predictors of bloodstream infection and its impact on mortality in septic arthritis: A 15-year review

**DOI:** 10.1093/ofid/ofae631.302

**Published:** 2025-01-29

**Authors:** Yongseop Lee, Yong Chan Kim, Jaeeun Seong, Sangmin Ahn, Min Han, Jung Ah Lee, Jung Ho Kim, Jin Young Ahn, Nam Su Ku, Jun Yong Choi, Joon-sup Yeom, Su Jin Jeong

**Affiliations:** Division of Infectious Diseases, Department of Internal Medicine and AIDS Research Institute, Yonsei University College of Medicine, Seodaemun-gu, Seoul-t'ukpyolsi, Republic of Korea; Department of Internal Medicine, Division of Infectious disease, Yongin Severance Hospital, Yonsei University College of Medicine, Yongin, Kyonggi-do, Republic of Korea; Division of Infectious Diseases, Department of Internal Medicine and AIDS Research Institute, Yonsei University College of Medicine, Seodaemun-gu, Seoul-t'ukpyolsi, Republic of Korea; Yonsei University College of Medicine, seoul, Seoul-t'ukpyolsi, Republic of Korea; Yonsei University School of Medicine, Seoul, Seoul-t'ukpyolsi, Republic of Korea; Yonsei University College of Medicine, seoul, Seoul-t'ukpyolsi, Republic of Korea; Yonsei University College of Medicine, seoul, Seoul-t'ukpyolsi, Republic of Korea; Yonsei University College of Medicine, seoul, Seoul-t'ukpyolsi, Republic of Korea; Division of Infectious Diseases, Department of Internal Medicine, Yonsei University College of Medicine, Seoul, Seoul-t'ukpyolsi, Republic of Korea; Yonsei University College of Medicine, seoul, Seoul-t'ukpyolsi, Republic of Korea; Division of Infectious Diseases, Department of Internal Medicine, Yonsei University College of Medicine, Seoul, Seoul-t'ukpyolsi, Republic of Korea; Yonsei University College of Medicine, seoul, Seoul-t'ukpyolsi, Republic of Korea

## Abstract

**Background:**

Septic arthritis is often complicated by bloodstream infection (BSI), which can lead to metastatin infections and sepsis. In this study, we aimed to identify risk factors for septic arthritis-related BSI and assess its impact on clinical outcomes.

**Table 1.**
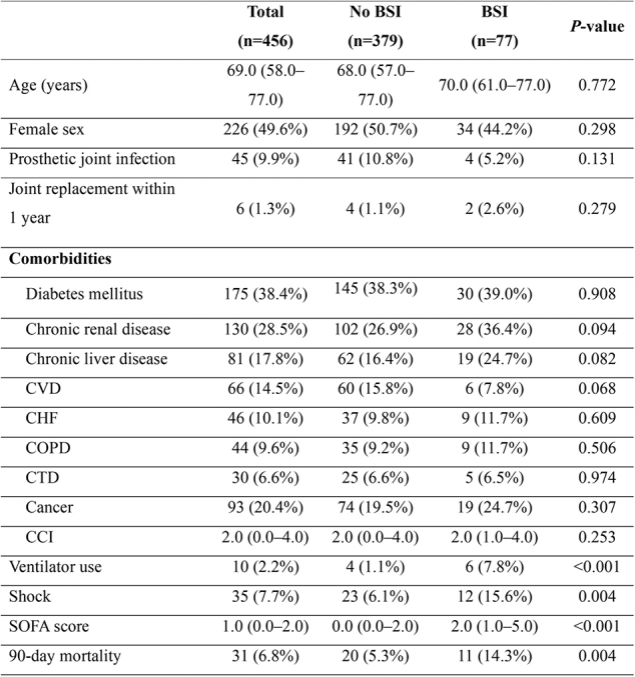
Comparison of clinical characteristics of septic arthritis patients with and without bloodstream infection.

**Methods:**

A retrospective review spanning 15 years (January 2009 to May 2023) was conducted on patients diagnosed with septic arthritis. Data from patients with positive synovial fluid cultures were analyzed.

**Table 2.**
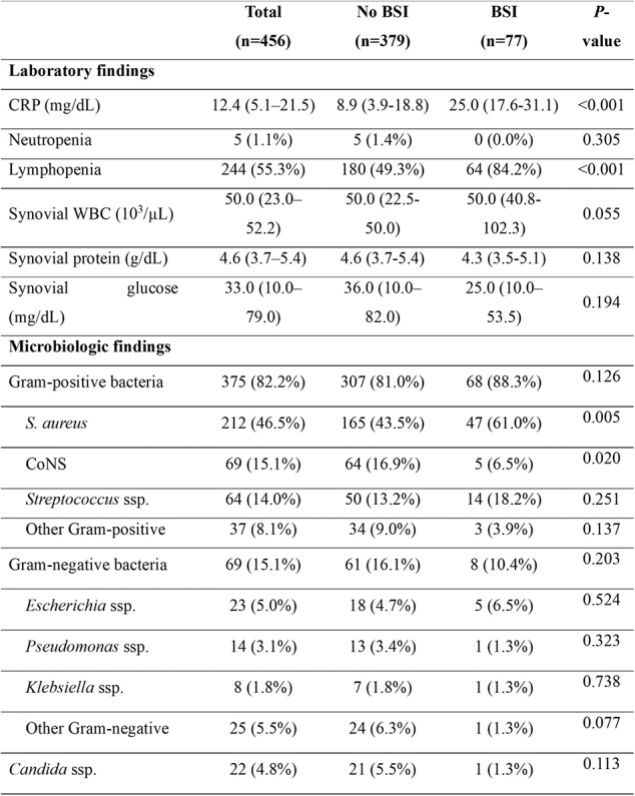
Laboratory and microbiologic findings of patients with septic arthritis according to bloodstream infections.

**Results:**

Among 456 patients with septic arthritis, 16.8% (n=77) developed BSI. The 90-day mortality rate was significantly higher in patients with BSI compared with those without BSI (14.3% vs. 5.3%, P=0.004). *Staphylococcus aureus* was the most commonly identified organism in synovial fluid cultures and was associated with an increased risk of BSI (adjusted odds ratio [aOR], 2.20; 95% confidence interval [CI], 1.15-4.34; P=0.019). Independent risk factors for BSI included higher Sequential Organ Failure Assessment (SOFA) score (aOR, 1.23; 95% CI, 1.06–1.44; P=0.009), lymphopenia (aOR, 2.84; 95% CI, 1.38–6.15; P=0.006), and elevated C-reactive protein (CRP, mg/dL) levels (aOR, 1.07; 95% CI, 1.05–1.10; P< 0.001). Age ≥70 years (aOR, 3.96; 95% CI, 1.49–11.85; P=0.009) and higher SOFA score (aOR, 1.36; 95% CI, 1.12–1.67; P=0.002) were significant predictors of 90-day mortality, although BSI itself was not.

**Table 3.**
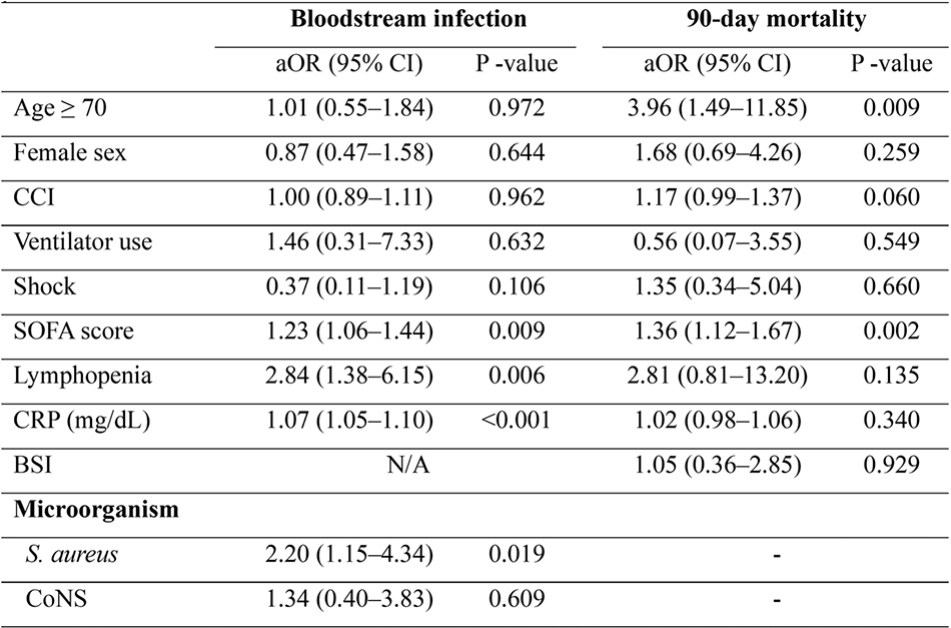
Multivariable analysis of risk factors for bloodstream infection and 90-day mortality in patients with septic arthritis. Variables with a P-value <0.05 in the univariable analysis were included in the multivariable analysis.

**Conclusion:**

Mortality in patients with septic arthritis was primarily associated with systemic sepsis resulting from BSI rather than the BSI itself. Understanding the relationship between septic arthritis-related BSI and clinical outcomes can aid physicians in managing systemic infections and improving patient care.

**Figure 1.**
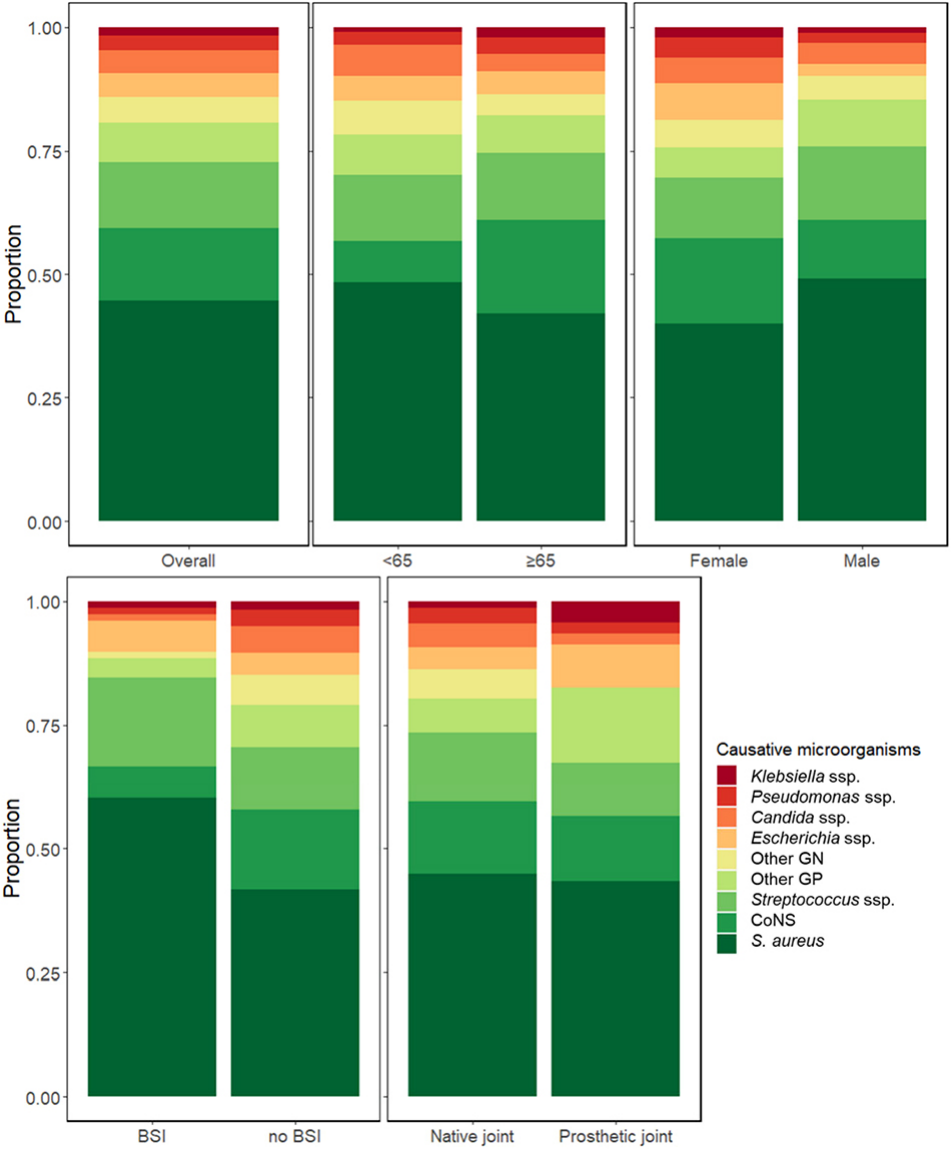
Distribution of causative organisms of septic arthritis.

**Disclosures:**

**All Authors**: No reported disclosures

